# Acetylation of glucosyltransferases regulates *Streptococcus mutans* biofilm formation and virulence

**DOI:** 10.1371/journal.ppat.1010134

**Published:** 2021-12-03

**Authors:** Qizhao Ma, Yangyang Pan, Yang Chen, Shuxing Yu, Jun Huang, Yaqi Liu, Tao Gong, Jing Zou, Yuqing Li

**Affiliations:** 1 State Key Laboratory of Oral Diseases, National Clinical Research Center for Oral Diseases, West China Hospital of Stomatology, Sichuan University, Chengdu, China; 2 Department of Pediatric Dentistry, West China Hospital of Stomatology, Sichuan University, Chengdu, China; Lunds universitet Medicinska fakulteten, SWEDEN

## Abstract

Lysine acetylation is a frequently occurring post-translational modification (PTM), emerging as an important metabolic regulatory mechanism in prokaryotes. This process is achieved enzymatically by the protein acetyltransferase (KAT) to specifically transfer the acetyl group, or non-enzymatically by direct intermediates (acetyl phosphate or acetyl-CoA). Although lysine acetylation modification of glucosyltransferases (Gtfs), the important virulence factor in *Streptococcus mutans*, was reported in our previous study, the KAT has not been identified. Here, we believe that the KAT ActG can acetylate Gtfs in the enzymatic mechanism. By overexpressing 15 KATs in *S*. *mutans*, the synthesized water-insoluble extracellular polysaccharides (EPS) and biofilm biomass were measured, and KAT (*actG*) was identified. The in-frame deletion mutant of *actG* was constructed to validate the function of *actG*. The results showed that *actG* could negatively regulate the water-insoluble EPS synthesis and biofilm formation. We used mass spectrometry (MS) to identify GtfB and GtfC as the possible substrates of ActG. This was also demonstrated by *in vitro* acetylation assays, indicating that ActG could increase the acetylation levels of GtfB and GtfC enzymatically and decrease their activities. We further found that the expression level of *actG* in part explained the virulence differences in clinically isolated strains. Moreover, overexpression of *actG* in *S*. *mutans* attenuated its cariogenicity in the rat caries model. Taken together, our study demonstrated that the KAT ActG could induce the acetylation of GtfB and GtfC enzymatically in *S*. *mutans*, providing insights into the function of lysine acetylation in bacterial virulence and pathogenicity.

## Author summary

Lysine acetylation is a regulatory post-translational modification (PTM) important in physiological processes across all domains of life. Although it has been well studied and characterized in eukaryotes, new insights into the lysine acetylation in bacteria have gained momentum in recent years, and hundreds to thousands of protein acetylation processes have been identified in various bacteria with novel enrichment strategies. However, the specific mechanisms of regulating lysine acetylation and function are still poorly understood. Therefore, we screened for the KAT mediating Gtfs acetylation by constructing 15 strains of *S*. *mutans* that overexpressed the GCN5-related *N*-acetyltransferases (GNAT) family members. Eventually, we identified and characterized ActG, a GNAT family member, that could catalyze the acetylation of GtfB and GtfC in *S*. *mutans* by the enzymatic mechanism, inversely related to their enzymatic activities, subsequently affecting the water-insoluble EPS synthesis and biofilm formation. In addition, ActG impaired the cariogenicity of *S*. *mutans* in a rat caries model. Thus, this study provides significant insights into the effect of lysine acetylation on *S*. *mutans* virulence and pathogenicity by regulating target protein functions and relative physiological processes.

## Introduction

Dental caries is a diet-biofilm-dependent disease in which biofilms formed by cariogenic and commensal microbes on tooth surfaces and played a vital causative role [1]. Over 700 different bacterial species have been identified in dental biofilms [2,3]. Among cariogenic bacteria, *Streptococcus mutans* is considered the principal etiologic agent with important virulence factors, such as acidogenicity, aciduricity, and biofilm formation. As the initiating factor, the biofilm lays the foundation for the pathogenesis of dental caries. On the one hand, biofilms provide a platform for pathogenic bacteria to colonize and accumulate in this multicellular cluster. On the other hand, they protect the bacteria within them and trap acids close to the tooth surface, further facilitating the development of carious lesions [4].

The extracellular biofilm matrix formation is mainly managed by glucosyltransferases (Gtfs), which can catalyze sucrose to synthesize extracellular polysaccharides (EPS) and promote adhesion of *S*. *mutans* to tooth surfaces [5]. *S*. *mutans* secretes three kinds of Gtfs (GtfB, GtfC, and GtfD), each synthesizing a structurally distinct glucan from sucrose. GtfB (formally known as Gtf-I) synthesizes water-insoluble glucans rich in α-1,3-linkages. GtfC (known as Gtf-SI) produces a mixture of water-soluble and water-insoluble glucans containing large amounts of α-1,6-linkages. Finally, GtfD (Gtf-S) predominantly forms water-soluble glucans. GtfB and GtfC, in particular, modulate the initial microbial adherence and coherence by binding the salivary pellicle, further contributing to the physical integrity and stability of the extracellular matrix by synthesizing water-insoluble glucans [4]. Deleting GtfB and GtfC resulted in a nearly complete loss of the ability to form biofilms, and deletion of GtfB abolished the formation of microcolonies by *S*. *mutans* [6].

Lysine acetylation is a dynamic and evolutionarily conserved PTM found extensively in prokaryotes and eukaryotes [7]. Currently, protein lysine acetylation is finely tuned by two distinct mechanisms: non-enzymatic acetylation using acetyl phosphate (AcP) or acetyl-coenzyme A (Ac-CoA) directly as a donor of the acetyl group, or enzymatic acetylation by acetyltransferase using Ac-CoA as the acetyl group donor [8]. Three main superfamilies of acetyltransferases have been identified, including GNAT (GCN5-*N*-acetyltransferase) superfamily, p300/CBP (CREB-binding protein) superfamily, and MYST (MOZ, Ybf2/Sas3, Sas2, and Tip60) superfamily [9–11]. However, only the GNAT superfamily is found in both eukaryotes and prokaryotes and characterized based on the core GNAT structural domain, while p300/CBP and MYST superfamily are identified exclusively in eukaryotes [12]. By transferring a negatively charged acetyl group from donor to a positively charged lysine residue, lysine acetyltransferase (KAT) changes the substrate’s charge and/or conformation, which might subsequently play an essential regulatory role in protein activity and stability [13,14].

To date, several global acetylome analyses of lysine acetylation have been reported in bacteria, including *Escherichia coli*, *Salmonella enteric*, *Bacillus subtilis*, *Porphyromonas gingivalis*, and *Aggregatibacter actinomycetemcomitans*, etc [15–18]. Our recent study showed that the acetylation levels of GtfB and GtfC in the *S*. *mutans* biofilm reduced by 29% and 22% compared with the planktonic condition, respectively [19]. However, it remains unknown whether KAT exists and catalyzes the acetylation of GtfB and GtfC, consequently affecting the biofilm formation in *S*. *mutans*.

In this study, to identify the possibility of KAT modifying GtfB and GtfC, we induced the overexpression of 15 genes whose products are annotated as GNATs in *S*. *mutans*. Further analysis demonstrated that one of the GNATs, ActG could catalyze the acetylation of GtfB and GtfC and impair their activities in the enzymatic mechanism, subsequently reducing the water-insoluble EPS production, biofilm formation, and caries formation in a rat caries model. In conclusion, these findings provide new insight into the regulatory mechanism of lysine acetylation on glucosyltransferase activities, with ActG possibly leading to potential therapies to inhibit the biofilm formation and cariogenicity of *S*. *mutans*.

## Results

### Identification of putative uncharacterized KAT modifying Gtfs

Compared with *S*. *mutans* in biofilm conditions, the acetylation level of Gtfs increased in planktonic conditions, and Gtfs acetylation inhibited their enzymatic activities [19]. However, the regulatory mechanism of Gtfs acetylation is still unknown in *S*. *mutans*. The *S*. *mutans* genome carries 15 genes whose products are annotated as GNATs (S1 Table). Therefore, we tested whether these uncharacterized KATs are involved in regulating Gtfs acetylation in *S*. *mutans*.

To determine whether these GNATs can affect Gtfs activities, we induced the overexpression of 15 GNATs in *S*. *mutans* and measured the biofilm biomass and water-insoluble EPS production. Compared with the parental strain, the biofilm biomass and water-insoluble EPS production decreased in these strains that overexpressed *actA*, *actG*, and *actH* (S1B and S1C Fig). Afterward, the growth of the four overexpression strains was examined in planktonic conditions. No significant impact on growth was observed in strains overexpressing *actG* and *actH* when grown in the brain heart infusion (BHI) broth. However, delayed growth was observed in strains overexpressing *actA* and *actL*, indicating that the reduction of EPS and biofilm in strains overexpressing *actA* or *actL* was due to a growth defect (S1A Fig). Therefore, *actG* and *actH* were selected to further identify the specific KAT modifying Gtfs. Then, the Gtfs acetylation levels and enzymatic activities were evaluated in strains overexpressing *actG* and *actH*, indicating that only in *actG* overexpressing strain, the Gtfs acetylation levels increased, and enzymatic activities were inhibited. Therefore, we selected ActG for further assessment of its ability to function as KAT modifying Gtfs.

### Overexpression of *actG* inhibited the water-insoluble EPS synthesis, biofilm formation, and Gtfs activities

**Fig 1:**
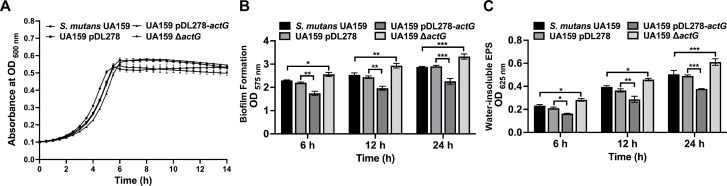
Effect of *actG* on bacterial growth, biofilm formation, and water-insoluble EPS synthesis in *S*. *mutans*. (A) Growth curves of *S*. *mutans* UA159, UA159 pDL278, UA159 pDL278*-actG*, and UA159 Δ*actG* in anaerobic condition for 12 h. (B, C) The biofilm biomass was determined by crystal violet staining assay (B) and anthrone-sulfuric acid method (C) when cultured in BHIS (1% sucrose wt/vol) in anaerobic condition for 6h, 12h, and 24h, respectively. Results are presented as mean ± SD (* *P* < 0.05, ** P < 0.01 or *** P < 0.001).

For better evaluation of the regulatory mechanism of ActG, the *actG* relative in-frame markerless deletion strain was successfully constructed. Compared with the parental strain, no significant growth differences were observed in UA159 pDL278*-actG* and Δ*actG* strains either at mid-logarithmic or stationary phases (Fig 1A). Then, the biofilm biomass and water-insoluble EPS synthesis were quantified using crystal violet dye staining and anthrone-sulfuric acid method, respectively. The results showed that the biofilm biomass and water-insoluble EPS synthesis decreased in the strain overexpressing *actG* and increased in the Δ*actG* strain compared with the parental strain UA159, respectively (Fig 1B and 1C).

**Fig 2:**
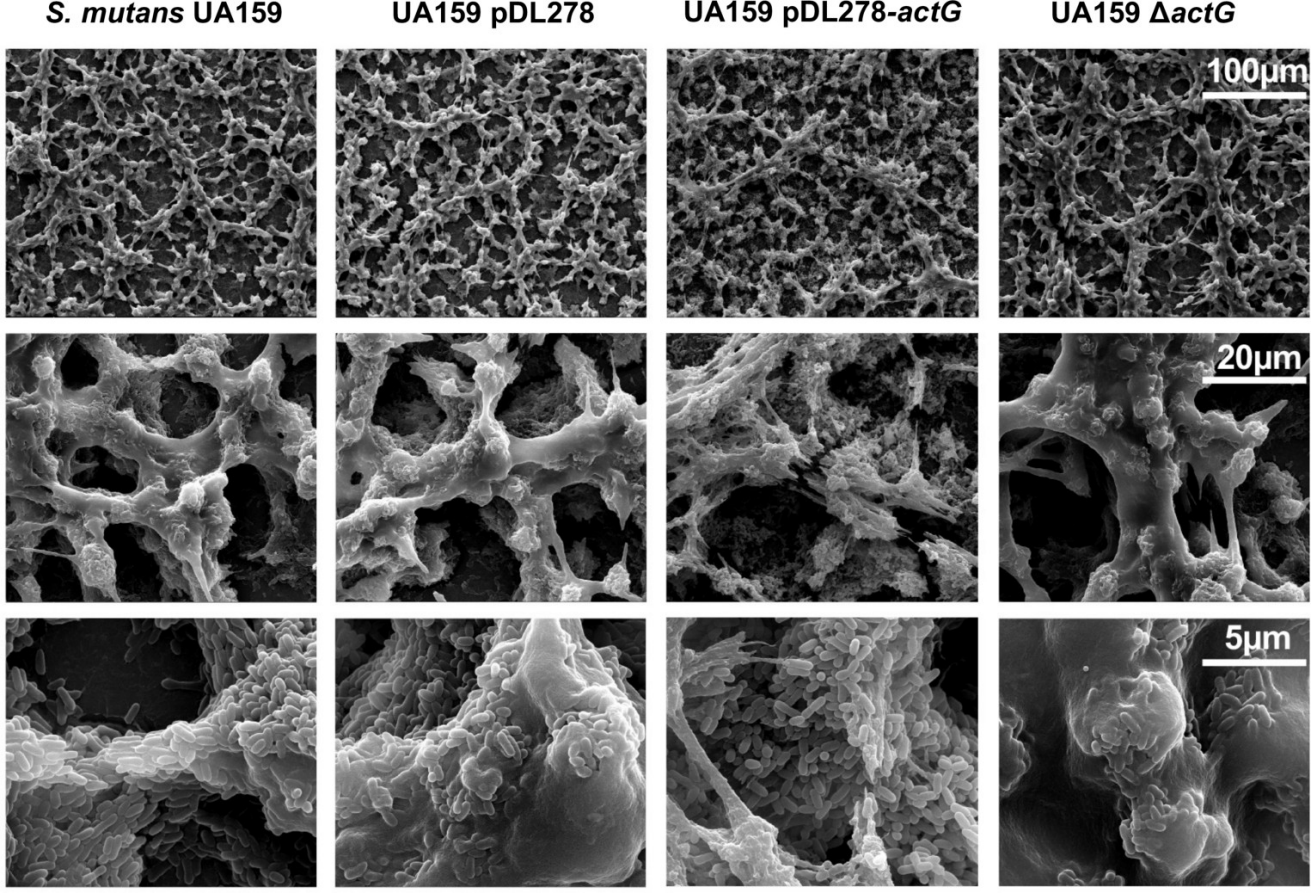
Effect of *actG* on biofilm architecture of *S*. *mutans*. Scanning electron microscopy analysis of the structure of 24-hours-biofilms. Images were captured at 1,000×, 5,000×, and 20,000× magnification. Representative images are shown from at least 5 randomly selected positions of each sample.

The impact of *actG* on the ability of *S*. *mutans* to form biofilm was further investigated by scanning electron microscopy (SEM). In the 24-hour-old biofilm, the strain overexpressing *actG* exhibited a relatively spongy and porous structure with decreased amounts of water-insoluble EPS. In contrast, Δ*actG* strains displayed a relatively dense and solid structure with increased amounts of EPS compared with the respective parental strain UA159 (Fig 2). In addition, no obvious abnormality was observed in cell morphology, including cell shape, cell wall, and cell swelling.

**Fig 3:**
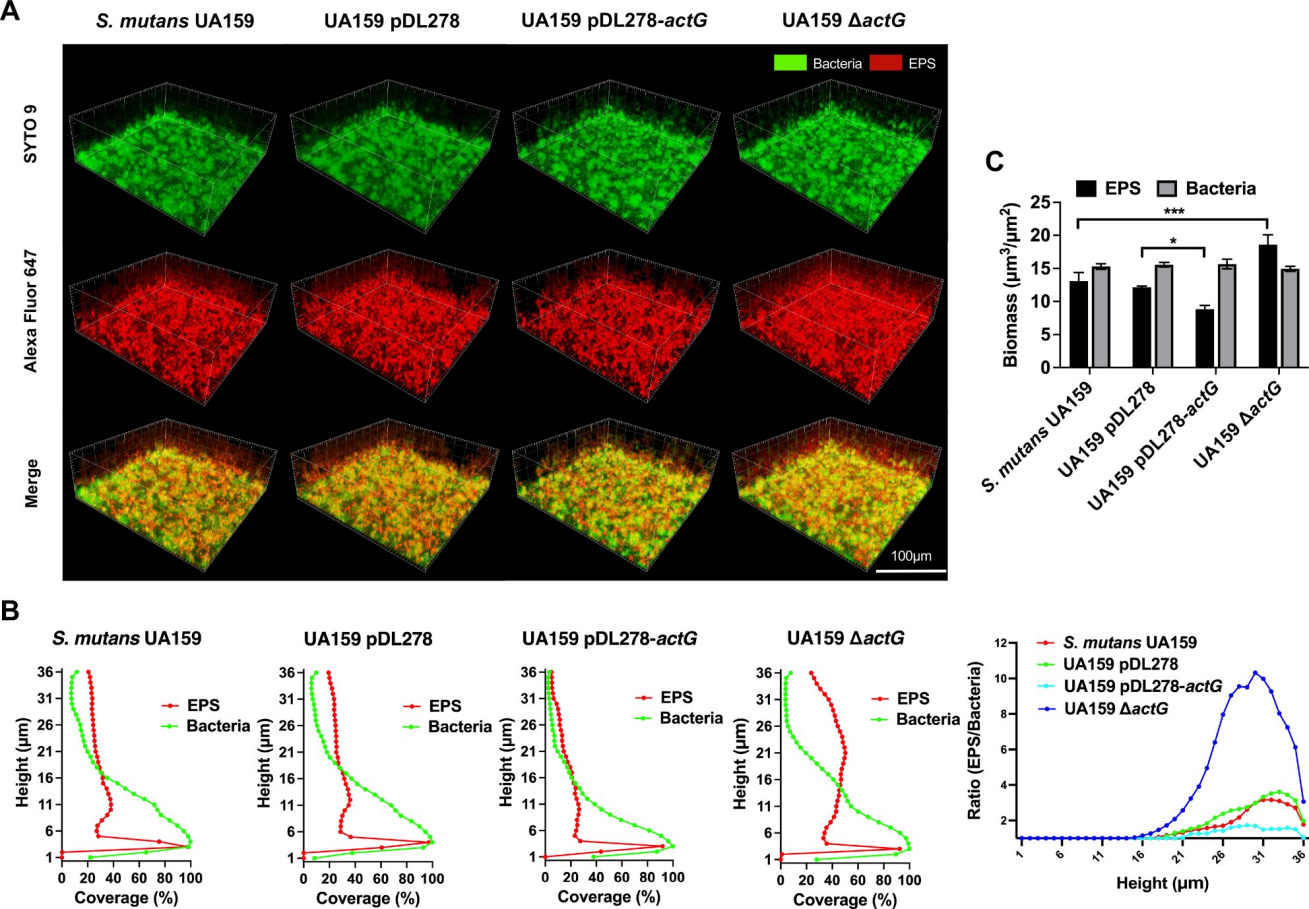
Effect of *actG* on biofilm structure and composition of *S*. *mutans*. Confocal laser scanning microscopy (CLSM) analysis of the three-dimensional visualization of bacteria (green) and EPS (red) of 24-hours biofilms. Images were captured at 60× magnification, and the three-dimensional reconstructions of biofilms were performed with IMARIS 7.0.0 (A). Quantitative analysis of bacteria and EPS biomass and the ratio of EPS to bacteria was performed with COMSTAT (B, C). Representative images are shown from at least 5 randomly selected positions of each sample. Results are presented as mean ± SD (* *P* < 0.05 or *** P < 0.001).

To further demonstrate the impact of *actG* on biofilm structure and water-insoluble EPS, confocal laser scanning microscopy (CLSM) was used. Fig 3A shows representative three-dimensional images of bacteria (green) and EPS (red) in 24-hour-old biofilms. In addition, the amounts of bacteria and EPS, the EPS/bacteria ratio, and the biofilm biomass from the glass coverslip surface to the liquid interface in three-dimensional vertical distribution were calculated using COMSTAT software (Fig 3B and 3C). The quantification data also confirmed that the strain overexpressing *actG* contained less water-insoluble EPS, and Δ*actG* strains contained more water-insoluble EPS than the parental strains pDL278 and UA159, respectively, and the bacterial counts remained consistent in different strains (Fig 3B). A similar consequence was observed in the EPS/bacteria ratio and biofilm biomass (Fig 3B and 3C).

**Fig 4:**
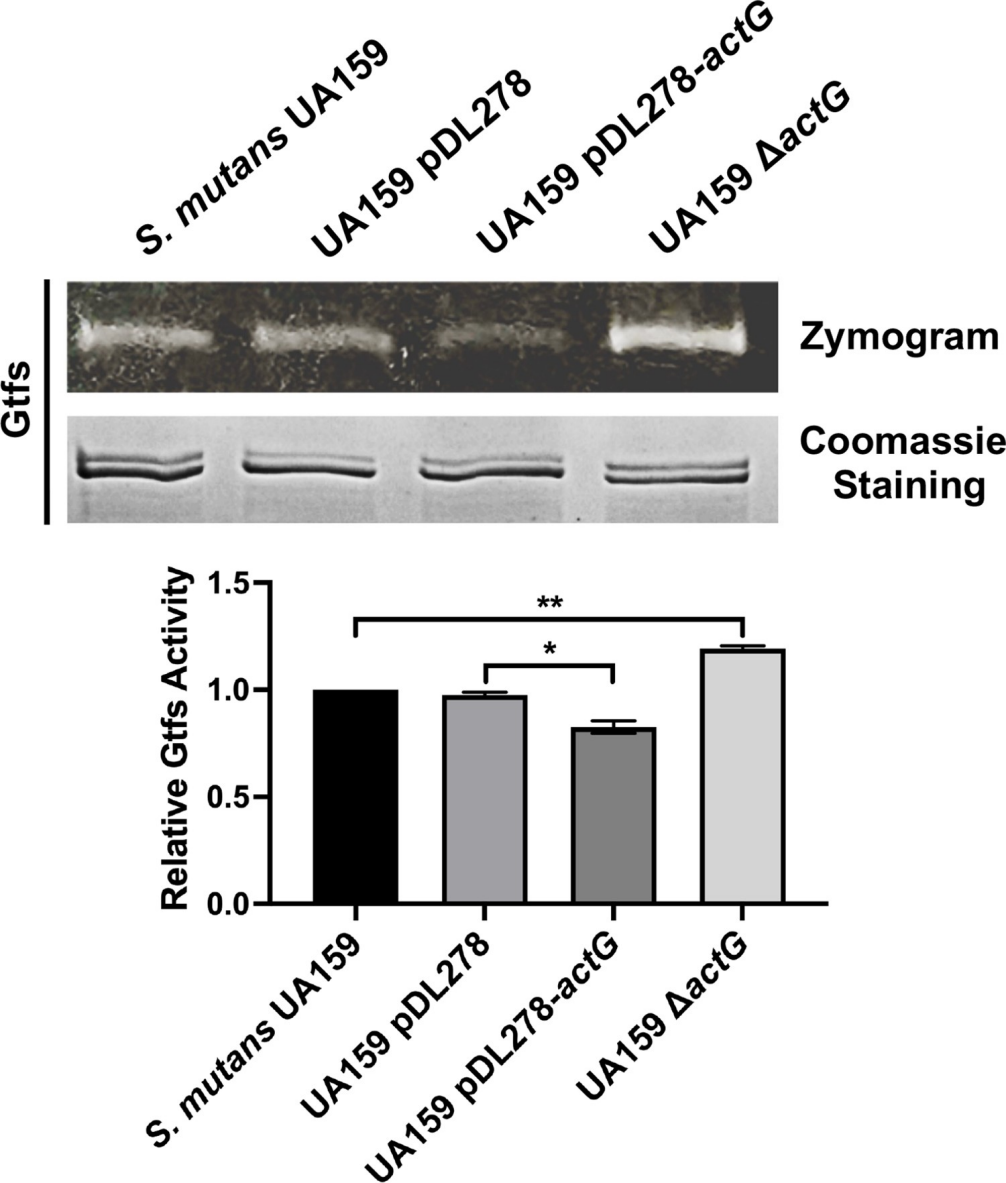
**Effect of *actG* on Gtfs activities of *S*. *mutans*.** Gtfs were analyzed for protein amount by Coomassie staining (upper and below) and glucan production by incubating gels with sucrose (upper and above), respectively. The band signals were quantified with Image J software and normalized to the control. Results are presented as mean ± SD (* *P* < 0.05 or ** P < 0.01).

The SDS-PAGE analysis and zymogram method were used to determine whether the reduction in water-insoluble EPS production was related to Gtfs activities. The upper band was GtfB (166 kDa) and GtfD (163 kDa), while the lower band was GtfC (153 kDa). Fig 4 (upper and below) shows that the protein amounts of Gtfs with two bands close to each other are consistent in different *S*. *mutans* strains. The Gtfs enzymatic activities were indicated by the brightness of the glucan bands. The Gtfs activities were significantly reduced in the strain overexpressing *actG*, and increased in Δ*actG* strains, compared with parental strains, respectively (Fig 4). These findings showed that *actG* reduced the amount of water-insoluble EPS and subsequently biofilm formation by affecting Gtfs activities.

### Identification of GtfB and GtfC as the substrates of ActG

**Fig 5:**
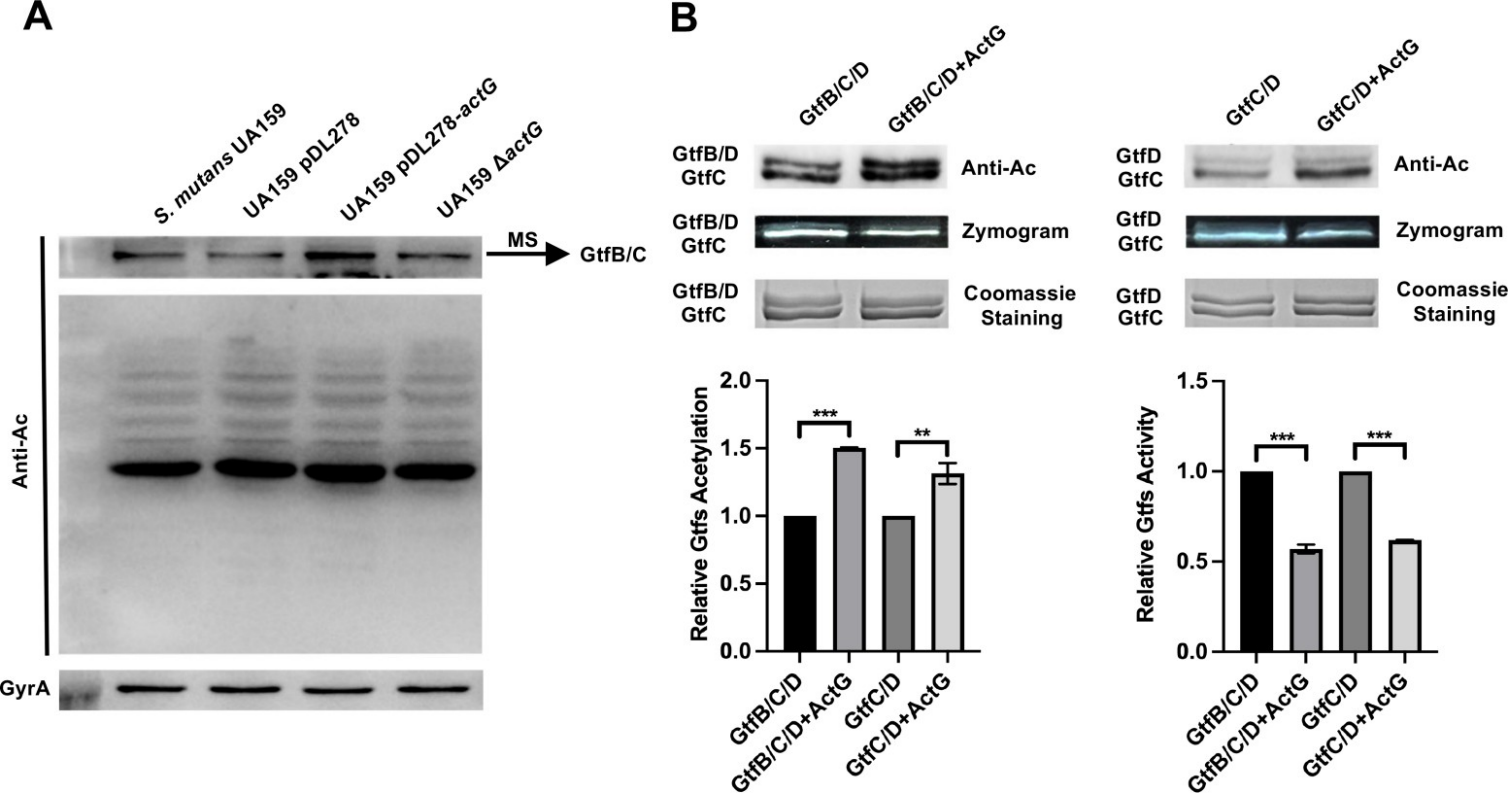
Effect of *actG* on total proteins acetylation and *in vitro* enzymatical acetylation. (A) The extracted total proteins were analyzed by anti-acetyl lysine Western blotting. The upregulated acetylated protein was identified by mass spectrometry (MS). (B) Coomassie staining, anti-acetyl lysine Western blotting, and zymogram analysis of Gtfs incubated with ActG as acetyltransferase and Ac-CoA as the acetyl donor for 3h at 37°C. The band signals were quantified with Image J software and normalized to the control. Results are presented as mean ± SD (** P <0.01 or *** P < 0.001).

We compared acetylation profiles of four different strains, including UA159, pDL278, pDL278*-actG*, and Δ*actG*, via anti-acetyl lysine Western blotting to determine the ActG target substrates. Indeed, we observed an upregulated acetylation band in the strain overexpressing *actG* compared with other strains (Fig 5A). Furthermore, we identified the upregulated acetylation band as GtfB and GtfC using mass spectrometry (MS) (S3 Table). These findings showed that GtfB and GtfC might be target substrates of ActG.

The purified ActG protein was incubated with GtfB and GtfC as the substrate and Ac-CoA as the acetyl donor to further explore whether ActG could induce the acetylation of GtfB and GtfC enzymatically. In addition, the GtfB and GtfC were incubated with Ac-CoA as the acetyl donor in a dose-dependent way to determine whether Ac-CoA alone can acetylate GtfB and GtfC non-enzymatically. The acetylation levels GtfB and GtfC were detected by anti-acetyl lysine Western blotting. As shown in S2 Fig, no significant differences in the acetylation levels of GtfB and GtfC were observed in the presence of Ac-CoA (0.5 mM) and the absence of ActG. However, enzymatically, the acetylation levels of GtfB and GtfC significantly increased when incubated with ActG at the Ac-CoA concentration of 0.5 mM for 3 h compared with the control (Fig 5B). Consistently, the activities of GtfB and GtfC significantly decreased under the same conditions (Fig 5B). These findings indicate that overexpression of *actG* reduced the Gtfs activities through acetylation modification, consistent with the previous phenotype.

### Different Gtfs activities and acetylation levels in clinically isolated strains

**Fig 6:**
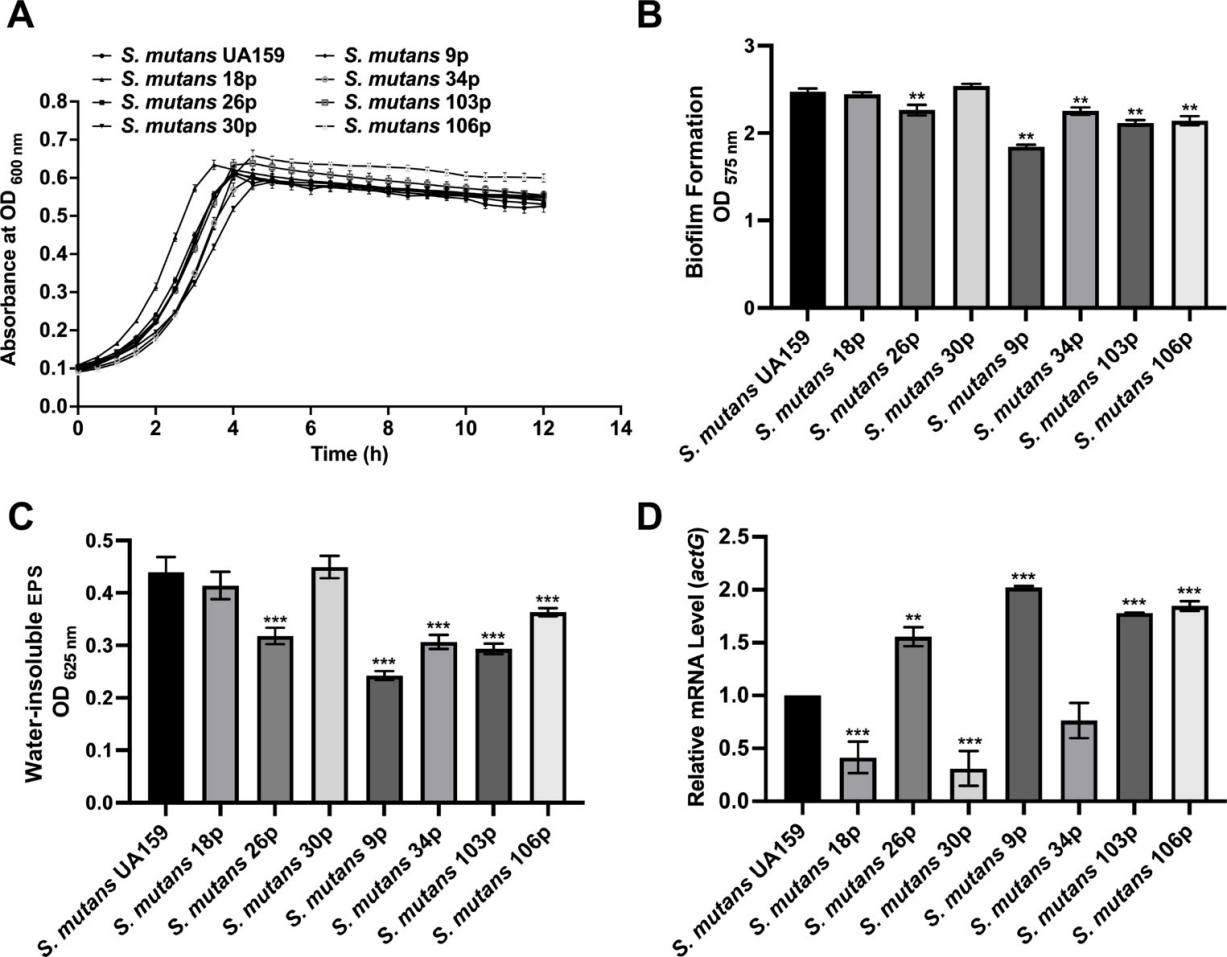
Effect of *actG* on clinically isolated strain*s*. (A) Growth curves of *S*. *mutans* UA159 and clinically isolated strains in anaerobic condition for 12 h. (B, C)The biofilm biomass was determined by crystal violet staining assay (B) and anthrone-sulfuric acid method (C) when cultured in BHIS (1% sucrose wt/vol) in anaerobic condition for 24h. (D) Gene expression level of *actG* in clinically isolated strains was determined by quantitative RT-PCR and calculated using the 2^-ΔΔCt^ method with values normalized to the reference gene 16S rRNA. Results are presented as mean ± SD (** P < 0.01 or *** *P* < 0.001).

We carried out follow-up experiments using seven clinical strains isolated from ECC children’s dental plaques to explore whether the expression of *actG* has a regulatory effect on Gtfs acetylation levels and activities and the water-insoluble EPS synthesis and biofilm biomass in clinical strains. We first monitored the growth of different clinically isolated strains in the BHI medium. As shown in Fig 6A, the growth rates of isolated strains were different at the mid-logarithmic phase; however, no difference was observed at the stationary phase. In the 24-hour-old biofilm, the biofilm biomass and water-insoluble EPS synthesis were calculated using crystal violet dye staining and anthrone-sulfuric acid method, respectively. The results showed that biofilm biomass and water-insoluble EPS reduced markedly in strains 26p, 9p, 34p, 103p, and 106p, compared to *S*. *mutans* UA159 (Fig 6B and 6C). By contrast, the CFU counts were the same in different strains, indicating that the ability to form biofilm and synthesize water-insoluble EPS was differently regulated in these strains (S3 Fig).

**Fig 7:**
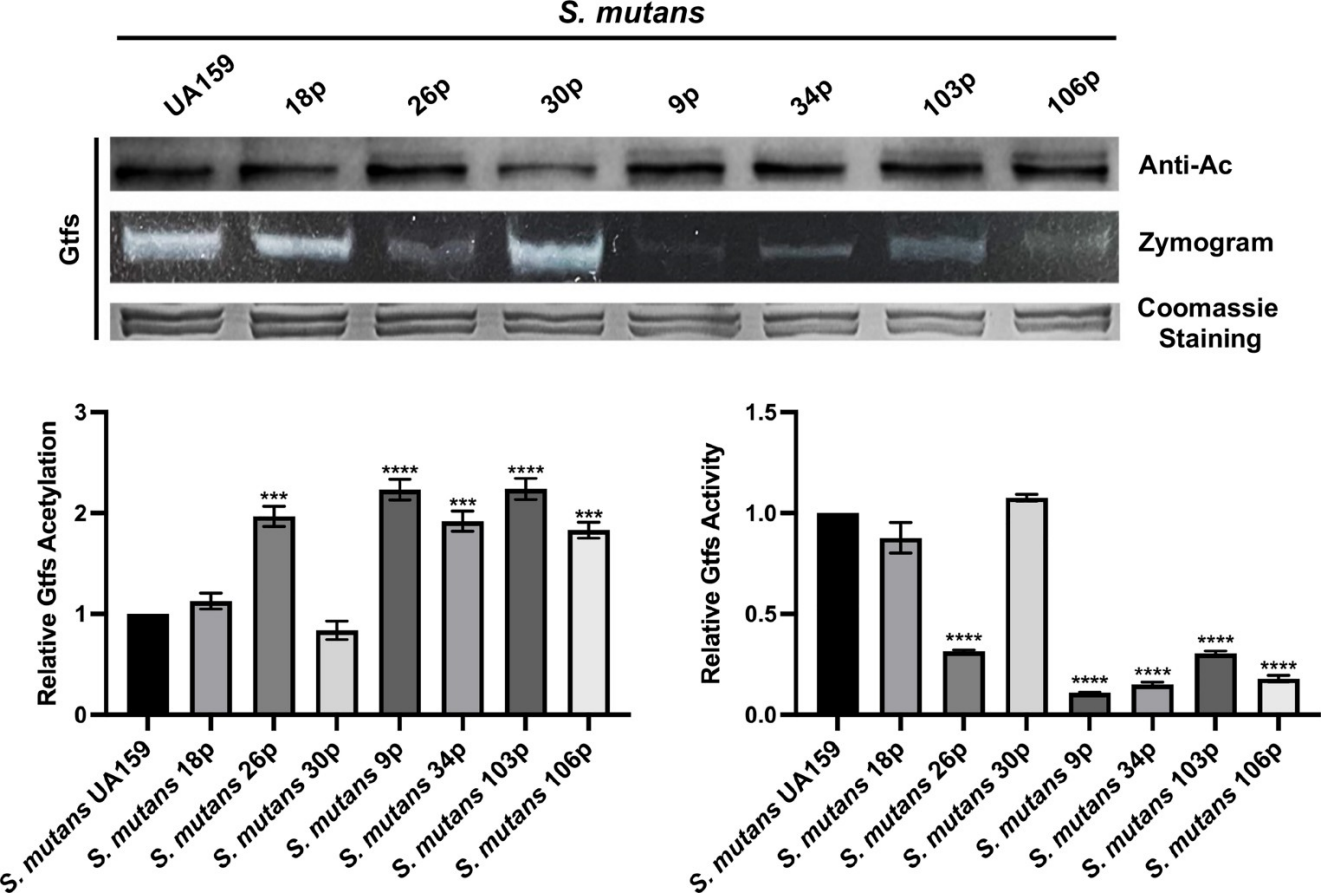
Effect of *actG* on the acetylation level and enzyme activity of Gtfs in clinically isolated strains. The extracted proteins were analyzed by Coomassie staining, anti-acetyl lysine Western blotting, and zymogram analysis. The band signals were quantified with Image J software and normalized to the control. Results are presented as mean ± SD (*** *P* < 0.001 or **** P < 0.0001).

The acetylation levels and activities of GtfB and GtfC in *S*. *mutans* clinical strains were examined to clarify the mechanism. As shown in Fig 7, the acetylation levels of Gtfs were enhanced in strains 26p, 9p, 34p, 103p, and 106p, compared to the UA159 strain. Furthermore, the activities of Gtfs were inversely related to the acetylation levels in the corresponding strains (Fig 7). We further quantified the expression of *actG* using RT-qPCR in different strains. The mRNA levels were consistent with the acetylation levels, except for samples 18p and 34p (Fig 6D).

### *actG* overexpression impaired the cariogenicity of *S*. *mutans* in rat caries models

**Fig 8:**
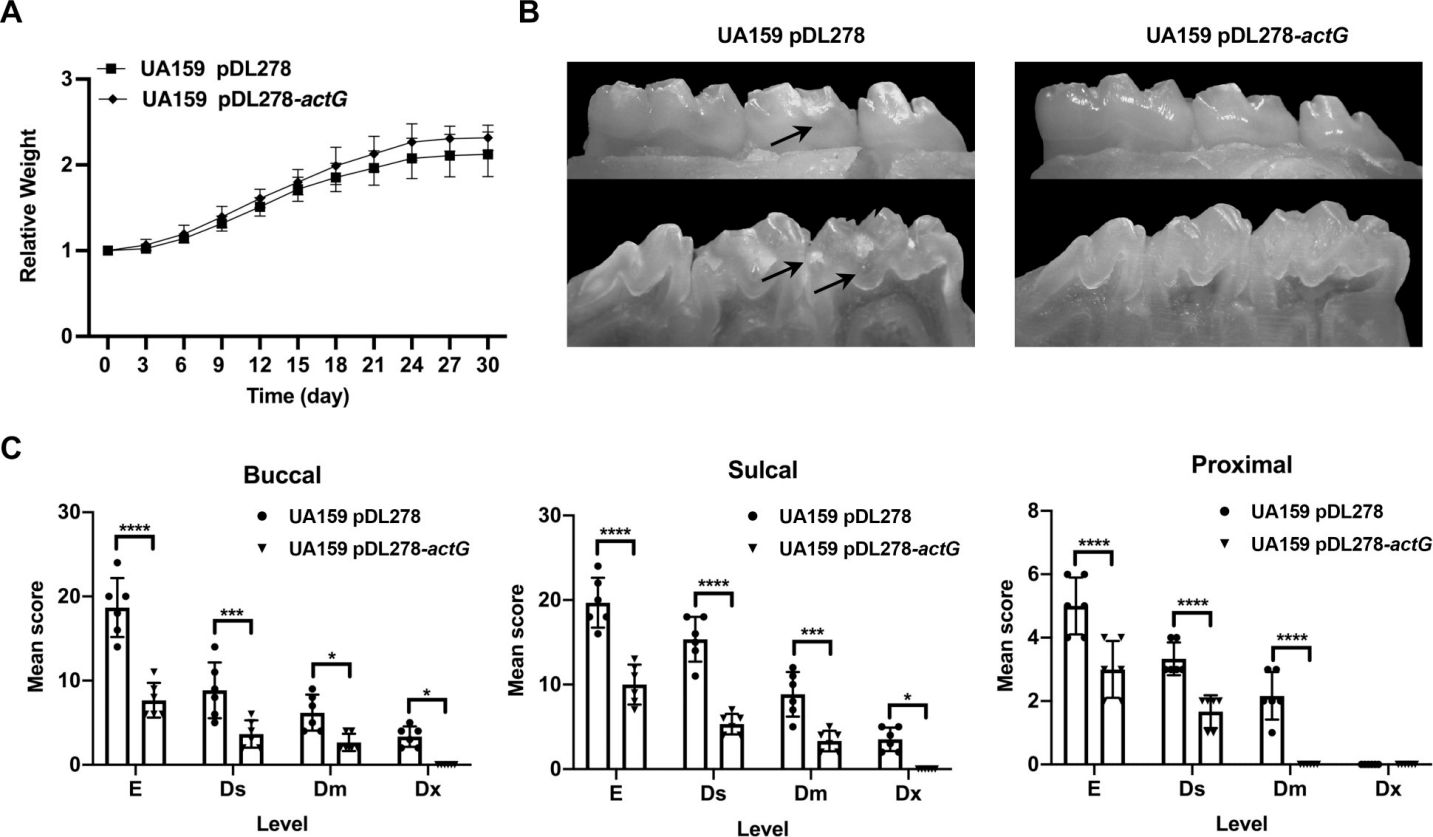
Effect of *actG* on the cariogenicity of *S*. *mutans* in rats. (A) The rats were weighed every three days until the termination of the experiment. (B) The representative images of the buccal (above panel) and sulcal (below panel) of the mandible with molars were displayed. The arrows indicate the representative carious lesions. (C) A statistic chart of the Keyes’ score was shown in Table 1. Each plot represents the caries score of each rat (n = 6) on buccal, sulcal, and proximal surfaces, respectively. The severity of carious lesions is classified into four kinds: enamel only (E), slight dentinal (Ds), moderate dentinal (Dm), and extensive dentinal (Dx). Results are presented as mean ± SD (* *P* < 0.05, ** P < 0.01, *** P < 0.001 or **** P < 0.0001).

The cariogenicity of UA159 pDL278 and UA159 pDL278*-actG* strains was compared in a rat caries model to evaluate the cariogenic effect of *actG in vivo*. All the rats remained in apparently good health throughout the study, with no significant difference in weight gain in either control or experimental groups (Fig 8A). The linear extent in one plane and the depth of penetration of carious lesions were evaluated by the Keyes’ score, including enamel only (E), slight dentinal (Ds), moderate dentinal (Dm), and extensive dentinal (Dx) [20]. The results showed that the cariogenicity was significantly impaired in the *actG* overexpressing strain compared with the UA159 pDL278 strain (Table 1). As shown in Fig 8B and 8C, the Keye’s scores of UA159 pDL278*-actG* group on the buccal, sulcal, and proximal surface significantly reduced compared to the UA159 pDL278 group. In addition, the carious lesion on the sulcal surface was more serious than the buccal surface either in the UA159 pDL278 or UA159 pDL278*-actG* group (Fig 8C). Concerning carious lesion severity, while observed in the UA159 pDL278 group, no Dx carious lesions were observed in the UA159 pDL278*-actG* group. Overall, *actG* overexpression impaired the cariogenicity of *S*. *mutans in vivo*, indicating its crucial role in regulating the virulence of *S*. *mutans*.

**Table 1:** Effect of *actG* on the cariogenicity of *S*. *mutans* in rats (Keyes’ score).

Infecting strain	Buccal					Sulcal				Proximal			
	E	Ds	Dm	DX	E		Ds	Dm	DX	E	Ds	Dm	DX
**UA159 pDL278**	18.7 ± 3.5	8.8 ± 3.3	6.2 ± 2.1	3.3 ± 1.2	19.7 ± 2.9		15.3 ± 2.7	8.8 ± 2.6	3.5 ± 1.4	5.0 ± 0.9	3.3 ± 0.5	2.2 ± 0.8	0.0
**UA159 pDL278*-actG***	7.7 ± 2.1^a^	3.7 ± 1.6^b^	2.7 ± 1.0^d^	0.0^d^	10.0 ± 2.4^a^		5.3 ± 1.2^a^	3.3 ± 1.2^b^	0.0^d^	3.0 ± 0.9^a^	1.7 ± 0.5^a^	0.0^a^	0.0

Results are presented as mean ± SD (n = 6; ^a^ *P* < 0.0001, ^b^ *P* < 0.001, ^c^ *P* < 0.01, or ^d^ *P* < 0.05).

E: enamel only.

Ds: slight dentinal (1/4 of the dentin between the enamel and pulp chamber affected).

Dm: moderate dentinal (between 1/4 and 3/4 of the dentin affected).

Dx: extensive dentinal (beyond 3/4 of the dentin affected).

## Discussion

Caries is a biofilm-related disease in which *S*. *mutans*, as the main cariogenic bacterial species, synthesizes water-soluble and water-insoluble EPS via Gtfs to facilitate biofilm formation and stabilization [21]. The biofilm protects the microorganisms from environmental stresses, enabling the formation of acidic microenvironments, promoting tooth demineralization, and forming carious lesions [22]. The gene expression regulation of *gtfs* has properly been explored at the transcriptional level in *S*. *mutans*, including the transcription factor exopolysaccharide synthesis regulator (EpsR), the glycosyltransferase SMU_833, and various inhibitors, indicating that targeting *gtfs* can efficiently reduce caries initiation and development via preventing EPS synthesis [23–25]. Nevertheless, the study of Gtfs activities regulation is reported rarely and mainly focuses on structure-based small molecules. Our previous study investigated the regulatory mechanism of lysine acetylation, an important PTM, on Gtfs activities in *S*. *mutans*. We found that Gtfs could be acetylated, and its acetylation in the biofilm was higher than in the planktonic condition [19]. However, the specific regulatory mechanism of Gtfs acetylation is still unknown. In this study, we identified a novel KAT, namely ActG, that could catalyze GtfB and GtfC acetylation modification enzymatically, thereby inhibiting their enzymatic activities, which reduced the water-insoluble EPS synthesis and biofilm formation of *S*. *mutans in vitro* and *in vivo*.

Lysine acetylation is ubiquitous in bacteria, and hundreds of acetylated proteins are identified by acetylome analysis [19,26–32]. Our previous study showed that Gtfs, the critical enzymes in biofilm formation, could be acetylated in *S*. *mutans*. Lysine acetylation can occur via two distinct mechanisms: enzymatic acetylation and non-enzymatic acetylation (chemical acetylation). The enzymatic mechanism is conducted by KAT that catalyzes the transfer of an acetyl group from Ac-CoA to the target substrate. Acetylation is mainly carried out in prokaryotes by GANT family members, such as Pat, YfiQ, MbtA, NhoA, and others [33–36]. The present study showed that overexpressing *actG* (one member in the GNAT family) in *S*. *mutans* could increase the acetylation of Gtfs. It further identified the GtfB and GtfC as the possible substrates of ActG by MS analysis (Fig 5A). In addition, it also demonstrated that ActG could upregulate the acetylation levels of GtfB and GtfC with Ac-CoA (0.5 mM) as the acetyl donor (Fig 5B). It is worth noting that the non-enzymatic mechanism has been identified in bacteria, where Ac-CoA could directly serve as the acetyl donor. For example, the mitochondria protein lysine acetylation can be facilitated by Ac-CoA in the mitochondrial matrix [37]. *B*. *subtilis* Ac-CoA synthetase (*Bs*AcsA) can be acetylated *in vitro* non-enzymatically by Ac-CoA [38]. Therefore, we determined the acetylation levels of GtfB and GtfC when they were incubated with Ac-CoA as the acetyl donor. The results showed no significant differences in the acetylation levels of GtfB and GtfC in 0.5-mM Ac-CoA compared with the control (S2 Fig). These findings showed that ActG could catalyze the lysine acetylation of GtfB and GtfC in an enzymatic mechanism.

The acetylation of the lysine group within the target protein affects a wide variety of biological processes by affecting enzymatic activities, including the regulation of RNA metabolism, DNA transactions, and motility and cell shape [39]. Thus far, bacterial lysine acetylation has been shown to have inhibitory effects on enzymatic activities [39–41]. For example, the acetylation level of RprY can be enhanced by Pat, diminishing its ability to bind to promoter DNA of *nqrA* and functionally impairing survival in oxidative stress in *P*. *gingivalis* [42]. Acetylated RcsB, the response regulator in two-component signal transduction systems (TCS), inhibited its ability to activate *rprA* transcription in *E*. *coli* [43]. Similar inhibitory effects were observed in other studies, including the inhibition of *cpxP* transcription by acetylated RNA polymerase (RNAP), the polyamine toxicity by acetylated SpeG in *E*. *coli*, and DNA binding by acetylated HBsu in *B*. *subtilis* [44–46]. Concerning the inhibitory effects of acetylation modification, we found that the acetylated GtfB and GtfC inhibited their activities (Fig 5B). The inhibitory effect of ActG on GtfB and GtfC activities subsequently reduced the water-insoluble EPS synthesis and biofilm formation. Similar results were reported on inhibiting Gtfs activities, resulting in decreased EPS synthesis and biofilm formation [47–49]. In addition, we found that *actG* did not affect the growth characteristics of *S*. *mutans* either at the mid-logarithmic phase or stationary phase, consistent with a previous study indicating that deletion of *Gtfs* reduced poor biofilm but did not delay or alter planktonic *S*. *mutans* growth [6].

Early childhood caries (ECC) is the most common biofilm-dependent childhood disease and a significant health problem worldwide [50]. It is characterized by *S*. *mutans* infection, which can utilize sucrose to synthesize EPS by Gtfs, contributing to the structural integrity of dental biofilm [51]. This study showed that the gene expression levels of *actG* were different in clinically isolated strains and subsequently affected the acetylation levels of GtfB and GtfC and their enzymatic activities (Figs 6 and 7). The findings in part explained the differences in the biofilm-forming capacity in clinically isolated *S*. *mutans* strains. In addition, the acetylation levels of GtfB and GtfC and gene expressions of *actG* did not exactly correspond in clinically isolated strains, indicating that there might be other regulatory mechanisms for Gtfs acetylation like deacetylases. Virulence differences between clinically isolated strains also were observed in phosphorylation modification. For example, the phosphorylation levels of CagA were distinct in clinically isolated strains of *Helicobacter pylori*, which is associated with gastric cancers [52]. A similar result was found in group A *Streptococcus*. Furthermore, the clinically isolated strains had different phosphorylation levels of CovR, which is responsible for virulence factor expression and bacterial invasiveness [53]. However, the mechanisms regulating *actG* expression in *S*. *mutans* have not yet been elucidated, and further explorations are necessary. Nevertheless, a subsequent rat caries model inoculated with the strain overexpressing *actG* confirmed the findings *in vivo*, and ActG could reduce the incidence and severity of dental caries by modifying lysine acetylation of Gtfs (Fig 8).

In conclusion, ActG, a GNAT family member that catalyzes the acetylation of GtfB and GtfC in *S*. *mutans* enzymatically, was identified, with an essential role in the water-insoluble EPS synthesis and biofilm formation. Further studies are underway to elucidate the complex regulatory mechanism of Gtfs acetylation, like deacetylation and the degradation of acetylated Gtfs. This study expands the understanding of lysine acetylation in *S*. *mutans* virulence and pathogenicity by regulating target protein functions and relative physiological processes. The present study revealed an important mechanism of Gtfs regulation by acetylation, suggesting that ActG might lead to potential therapies to prevent dental caries.

## Materials and methods

### Ethics statement

All rat experiments in this study were performed in accordance with the protocols and procedures approved by the Institutional Review Board of West China Hospital of Stomatology (WCHSIRB-D-2021-156, Sichuan University, Chengdu, China). The animal care and use protocol adhered to the Chinese National Laboratory Animal-Guidelines for Ethical Review of Animal Welfare. The ECC samples in this study were approved by the Institutional Review Board of West China Hospital of Stomatology (WCHSIRB-D-2015-084, Sichuan University, Chengdu, China).

### Bacterial strains and growth conditions

All the strains, plasmids, and primers used in this study are listed in the S1 and S2 Tables. *S*. *mutans* UA159 was commercially obtained from the American Type Culture Collection (ATCC, Manassas, VA, USA). The *gtf* mutants (Δ*gtfB*) of *S*. *mutans* were kindly provided by Robert A. Burne (Department of Oral Biology, University of Florida, Gainesville, FL). *S*. *mutans* UA159 and its derivatives were routinely grown in the BHI broth (Difco, Sparks, MD, USA) under anaerobic conditions (85% N_2_, 5% CO_2_, 10% H_2_) at 37°C. For the biofilm assay, 1% sucrose (Sigma, 1%, wt/vol) was added to the BHI medium (BHIS). In addition, *E*. *coli* and its derivatives were cultured in Luria-Bertani LB medium (BD, Sparks, MD, USA) aerobically (95% air, 5% CO_2_) with spectinomycin (1 mg/mL) or kanamycin (30 μg/mL), when necessary.

### Construction of overexpression strains

The genes of GNAT family members (ActA-ActO) were amplified from *S*. *mutans* genomic DNA by polymerase chain reaction (PCR). All the primers were designed using an online tool provided by TaKaRa (http://www.takarabio.com/US/Products/Cloning_and_Competent_Cells/Selection_Guides/Online_In-Fusion_Tools) listed in the S2 Table. The PCR products were purified and cloned into linearized *E*. *coli*-*Streptococcus* shuttle vector pDL278 at the same insertion site using an In-Fusion HD cloning kit (TaKaRa, Japan). Then, these recombined plasmids were respectively transformed into *S*. *mutans* UA159 to generate overexpression strains of GNAT family members, selected using plates containing spectinomycin (1 mg/mL) and verified by PCR and sequencing.

### Construction of markerless in-frame deletion mutants

The markerless in-frame deletion mutants of the *S*. *mutans* GNAT family member ActG and ActH were constructed using a previously described two-step transformation method [49]. All the primers designed in this study are listed in the S2 Table. First, approximately 1 kb of the homologous sequence upstream and downstream of the *actG* open reading frame was amplified from *S*. *mutans* UA159 genomic DNA using PCR with the upF/upR and dnF/dnR primers. Next, the selection cassette IFDC2 (positive for erythromycin and negative for *p*-Cl-Che) was amplified by the ldhF/ermR primers. The fragments containing the overlapping regions were then ligated using the overlap extension PCR with upF/dnR primers, transformed into *S*. *mutans* UA159, and selected using plates containing erythromycin (12 μg/mL). Concerning the second transformation, upstream and downstream fragments of *actG* were amplified using PCR with the upF/updnR and dnF/dnR primers and ligated using overlap extension PCR. The ligated fragment was transformed into the mutant strain containing IFDC2 and selected using plates containing *p*-Cl-Che (4 mg/mL). Finally, the markerless in-frame deletion mutant was confirmed by PCR and sequencing.

### Growth curve assay

Overnight cultures of *S*. *mutans* and its derivatives were subcultured at a dilution of 1:10 in BHI medium until an optical density of 0.5 was achieved at 600 nm (OD_600nm_). Then, the cultured cells were diluted by 1:100 and inoculated in a 96-well cell culture plate, and each well was covered by sterile mineral oil. The OD_600nm_ was measured by a spectrophotometer (BioTek, Winooski, VT, USA) every 30 min. Each experiment was performed with triplicate samples at each time interval. Thus, the results corresponded to three experiments independently.

### Biofilm formation assay and quantification of the water-insoluble EPS

Biofilm formation was used to provide an overall assessment of biofilm biomass by the crystal violet (CV) staining method. First, the diluted strains were anaerobically cultured in 24-well plates for 6, 12, and 24 h, respectively. After incubation, the growth medium was gently removed, and the biofilm was washed with sterile phosphate-buffered saline (PBS) three times and replaced with 0.01% CV for 15 min. Next, the wells were rinsed with PBS three times, and 33% acetic acid was added and incubated for 30 min. The absorbance of the solution was measured at OD_575nm_ by a spectrophotometer.

After incubation under the same conditions with biofilm formation assay, the culture fluid was gently removed, and the biofilm was washed with sterile PBS three times. The cells in each well were resuspended by vigorous pipetting and vertexing and harvested in a 1.5-mL centrifuge tube. After centrifuging at 6,000 ×g for 10 min at 4°C, the pellet was resuspended with 1-M NaOH solution and incubated at 37°C for 2 h. Finally, the alkali-soluble carbohydrate solution was reacted with three volumes of anthrone-sulfuric acid reagent at 95°C for 6 min. After natural cooling, the absorbance of the solution was measured at OD_625nm_ by a spectrophotometer. Each experiment was performed with triplicate samples at each time interval. Therefore, the results corresponded to three experiments independently.

### SEM and CLSM analysis of biofilms

The biofilm structure and the EPS production were observed by scanning electron microscopy (SEM). Initially, the diluted strains were anaerobically cultured in 24-well plates covered with glass coverslips at the bottom for 24 h. After incubation, the biofilm was rinsed with sterile PBS three times and fixed with 2.5% glutaraldehyde at 4°C overnight. The coverslips were rinsed with PBS once and serially dehydrated in ethanol. The samples were observed by SEM (FEI, Hillsboro, OR, USA).

The structural composition of the biofilm was observed and relatively quantified by *in situ* labeling of bacterial cells and EPS as described previously [54]. After incubation with Alexa Fluor 647-labeled dextran conjugate (1 μM; Life Technologies, Grand Island, NY, USA), the supernatant was discarded, and the biofilm was rinsed with sterile PBS three times. Then, the biofilm was labeled with SYTO 9 green-fluorescent nucleic acid stain (2.5 μM; Life Technologies). The labeled biofilm was captured with a confocal laser scanning microscope (Leica DM IRE2) with the ×60 oil immersion objective lens (CLSM; Leica, Wetzlar, Germany). Image gates were set at 655–690 nm for Alexa Fluor 647 and 495–515 for SYTO 9. The three-dimensional structure of the biofilm was reconstructed by IMARIS 7.0.0 (Bitplane, Zurich, Switzerland). COMSTAT image-processing software was used to analyze the confocal image stacks and quantify the biomass (coverage percentage) of bacterial cells and EPS. At least three independent experiments were performed, and the displayed images are representative.

### Purification of recombinant ActG

*actG* was amplified from *S*. *mutans* genomic DNA by PCR and purified, digested by *Nco*I and *Xho*I, and cloned into the expression vector pET28a (Novagen) with an N-terminal fusion of 6×His-tag. Next, the reconstructed plasmid was cloned to the *E*. *coli* BL21 (DE3) cells. Proteins were purified, and concentrations were determined as described previously [55]. First, overnight cultures of the transformant were diluted 1:20 with fresh LB medium containing 50 μg/mL of kanamycin until OD_600nm_ of 0.6 was achieved. After further growth with 1-mM isopropyl-β-D-thiogalactopyranoside (IPTG) that induced protein expression, the cell pellets were harvested and lysed by sonification. Then, the recombinant ActG was purified using a His-tagged protein purification kit (Beyotime, Shanghai, China) and concentrated by 5-kDa MWKO ultra-filtration (Millipore Amincon, Merck, Germany) from the cell debris. The purified ActG was confirmed by SDS-PAGE and stored at -80°C for future use.

### LC-MS/MS analysis

The tryptic peptides were dissolved in 0.1% formic acid (solvent A), directly loaded onto a homemade reversed-phase analytical column (15 cm in length, 75 μm i.d.). The gradient was comprised of an increase from 6% to 23% solvent B (0.1% formic acid in 98% acetonitrile) over 16 min, 23% to 35% in 8 min, and increasing to 80% in 3 min, followed by maintaining at 80% for the last 3 min, all at a constant flow rate of 400 nL/min on an EASY-nLC 1000 UPLC system. The peptides were subjected to NSI source, followed by tandem mass spectrometry (MS/MS) in Q Exactive Plus (Thermo), coupled online to the UPLC. The applied electrospray voltage was 2.2 kV. The m/z scan range was 350–1800 for a full scan, and intact peptides were detected in the Orbitrap at a resolution of 70,000. Peptides were then selected for MS/MS using the NCE setting at 28, and the fragments were detected in the Orbitrap at a resolution of 17,500. A data-dependent procedure that alternated between one MS scan was followed by 20 MS/MS scans with 15.0 s dynamic exclusion. Automatic gain control (AGC) was set at 5E4.

The collected MS/MS data were processed with Proteome Discoverer 2.1. Tandem mass spectra were searched against the *S*. *mutans* database. Trypsin/P was specified as a cleavage enzyme allowing up to two missing cleavages. The mass error was set to 10 ppm for precursor ions and 0.02 Da for fragment ions. Carbamidomethyl on Cys was specified as a fixed modification, and oxidation on Met was specified as a variable modification. Peptide confidence was set at high, and peptide ion score was set at >20.

### Western blotting

Western blotting was performed as described previously [56]. The protein concentrations were determined by the BCA protein assay kit (Beyotime) following the manufacturer’s instructions. Equal amounts of protein (30 μg) were mixed with SDS-PAGE sample buffer, boiled for 10 min, and separated using 8% SDS-PAGE (110 V). The polypeptides were electrophoretically transferred to PVDF membranes blocked in 5% (wt/vol) non-fat dry milk at room temperature for 1 h. Then, the membranes were incubated with anti-acetyl lysine (Anti-Ac) Rabbit antibody (PTM-105, PTM BIO, Hangzhou, China), which was diluted 1:1000 in TBST at 4°C overnight. After six times of washing with TBST, the Anti-Ac membranes were incubated with HRP-conjugated goat anti-rabbit secondary antibody at 1:10000 dilution in TBST at room temperature for 2 h. The membranes were visualized with the immobilon Western Chemiluminescent HRP substrate kit (Millipore). The images were captured with the BioRad GS-700 Imaging Densitometer and quantified using Image J software. Polyclonal antibodies to GyrA were used as a control for whole-cell extracts.

### Zymogram assay for Gtfs activities

Gtfs activities were analyzed by zymogram methods as described previously [47]. First, the proteins were dialyzed at 4°C against sodium phosphate buffer containing protease inhibitor and concentrated by the BSC protein assay kit. Then, the dialyzed proteins were boiled or not and separated by 12% SDS-PAGE. One was used for protein staining with Coomassie blue dye, while the other was subjected to zymogram assay. The latter followed the procedures that the gels were washed twice with renaturing buffer and then incubated with 0.2-M sodium phosphate buffer containing 0.2% dextran and 5% sucrose at 37°C overnight. Finally, the reactions were stopped by washing twice with distilled water at 4°C for 10 min. The images were captured using a digital camera and quantified by Image J software (Rawak software Inc, Stuttgart, Germany).

### *In vitro* acetylation analysis

The harvested Gtfs were incubated with Ac-CoA as the acetyl donor in 20-μL total reaction volume containing 100-mM Tris-HCl (pH = 7.5), 150-mM NaCl, and 10-mM MgCl_2_ in the presence or absence of ActG. Reactions occurred at 37°C for 3 h and stopped by adding SDS sample buffer or boiling in SDS sample buffer. Then, the samples were analyzed by SDS-PAGE, Western blotting, and zymogram method.

### ECC samples and CFU counts

The ECC samples were from our previous study [57], and detailed information on each sample can be found in the S4 Table. These isolated strains and standard strain UA159 were analyzed by experimental methods as described previously [58], using a primer set for *S*. *mutans* identification (Forward: 5’-GGCACCACAACATTGGGAAGCTCAGTT-3’, Reverse: 5’-GGAATGGCCGCTAAGTCAACAGGAT-3’). Concerning CFU counts, after resuspending cells from 24-hour biofilms, the cells were serially diluted 10^6^-fold, plated onto BHI agar plates, and incubated anaerobically at 37°C for 48 h before they were used to determine CFU counts.

### RNA extraction and qRT-PCR

Total RNA was extracted and purified as described previously [59]. First, RNA (1 μg) was reverse-transcribed to cDNA using the cDNA synthesis kit (TaKaRa, Shiga, Japan). Then, quantitative reverse transcriptase PCR (qRT-PCR) was performed using SYBR green master mix on the Bio-Rad CFX96 system (Bio-Rad). All the primers used in this study are listed in the S2 Table. Relative expression levels of genes were calculated using the 2^-ΔΔCt^ method, with values normalized to the reference gene 16S rRNA.

### Rat caries model

The cariogenicity of UA159 pDL278*-actG* was compared with UA159 pDL278 in the rat caries model as described previously [47,60]. Twelve female specific pathogen-free (SPF) Sprague Dawley (SD) rats (Chengdu Dossy Experimental Animals Co., Ltd, China) three weeks of age and weighing 50±5 g were purchased and randomly assigned to two groups (n = 6). The rats were given distilled water with ampicillin (1 g/kg) to drink for the first three days and distilled water without ampicillin for another day to elute the antibiotic. The rats were incubated with either UA159 pDL278 or UA159 pDL278*-actG* strain by oral swabbing with a fresh overnight culture for three consecutive days. In addition, the rats were provided with the cariogenic diet 2000 (*TrophicDiet*, Trophic Animal Feed, Suzhou, China) and sterile water containing 5% (wt/vol) sucrose. Oral swabs were taken five days after infection to confirm colonization. The rats were weighed every three days until the termination of the experiment. The rats were sacrificed three weeks after incubation, and their mandibles were obtained. After removing flesh from the jaws, all the molar teeth were stained with ammonium purpurate (0.4% concentration) for 6 h and then hemi-sectioned with a cutter. The images were captured under a stereomicroscope (Leica EZ4HD; Leica Microsystems AG, Heerbrugg, Switzerland) and scored for carious lesions by the Keyes method [20].

### Statistical analysis

Statistical analyses of the data were performed using SPSS 20.0 (SPSS Software Inc, Chicago, IL, USA) and Prism 9.0 (GraphPad Software Inc, San Diego, CA, USA). The Student’s *t*-test was used to compare data between two groups. One-way analysis of variance (ANOVA) and the Tukey test were performed to compare data between multiple groups. *P*<0.05 was considered statistically significant.

## Supporting information

S1 Fig Effect of overexpressing GNAT family genes on bacterial growth, biofilm formation, and water-insoluble EPS synthesis in *S*. *mutans*. (A) Growth curves of S. mutans UA159 and GNAT family genes overexpression strains in anaerobic conditions for 12 h. (B, C) The biofilm biomass was determined by crystal violet staining assay (B) and anthrone-sulfuric acid method (C) when cultured in BHIS (1% sucrose wt/vol) in anaerobic condition for 24 h. Results are presented as mean ± SD (* *P* < 0.05, ** *P* < 0.01, *** *P* < 0.001 or **** *P* < 0.0001).
(TIF)

S2 Fig *In vitro* non-enzymatical acetylation analysis. Coomassie staining and anti-acetyl lysine Western blotting analysis of GtfB/C/D (A) and GtfC/D (B) incubated with Ac-CoA as the acetyl donor at different concentrations for 3 h at 37°C. The band signals were quantified with Image J software and normalized to the control. Results are presented as mean ± SD (*P* > 0.05).
(TIF)

S3 Fig The colony-forming units (CFUs) from biofilms of clinically isolated strain*s*. The cells from 24-hours biofilms were plated onto the BHI agar plate, incubated anaerobically for 48 h at 37°C, and the CFUs were counted. Results are presented as mean ± SD (*P >* 0.05).
(TIF)

S1 Table Bacterial strains and plasmids in this study. (XLSX)

S2 Table Primers used in this study. (XLSX)

S3 Table Identified proteins in MS analysis. (XLSX)

S4 Table Information of each ECC clinically isolated sample. (XLSX)
