# Supplementary figures and images for: Acetylation of glucosyltransferases regulates Streptococcus mutans biofilm formation and virulence
Source: PLoS Pathog. 2021 Dec 3;17(12):e1010134. doi: 10.1371/journal.ppat.1010134 (PMC8673623; doi:10.1371/journal.ppat.1010134)

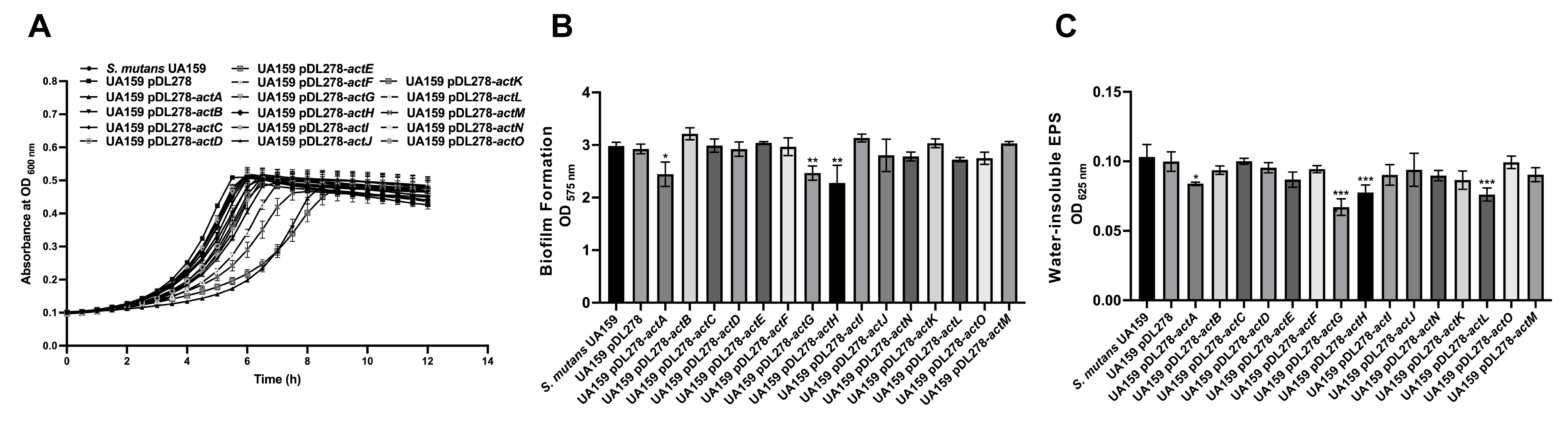

Supplement: S1 Fig — (A) Growth curves of S. mutans UA159 and GNAT family genes overexpression strains in anaerobic conditions for 12 h. (B, C) The biofilm biomass was determined by crystal violet staining assay (B) and anthrone-sulfuric acid method (C) when cultured in BHIS (1% sucrose wt/vol) in anaerobic condition for 24 h. Results are presented as mean ± SD (* P < 0.05, ** P < 0.01, *** P < 0.001 or **** P < 0.0001). (TIF) [file ppat.1010134.s001.tif]

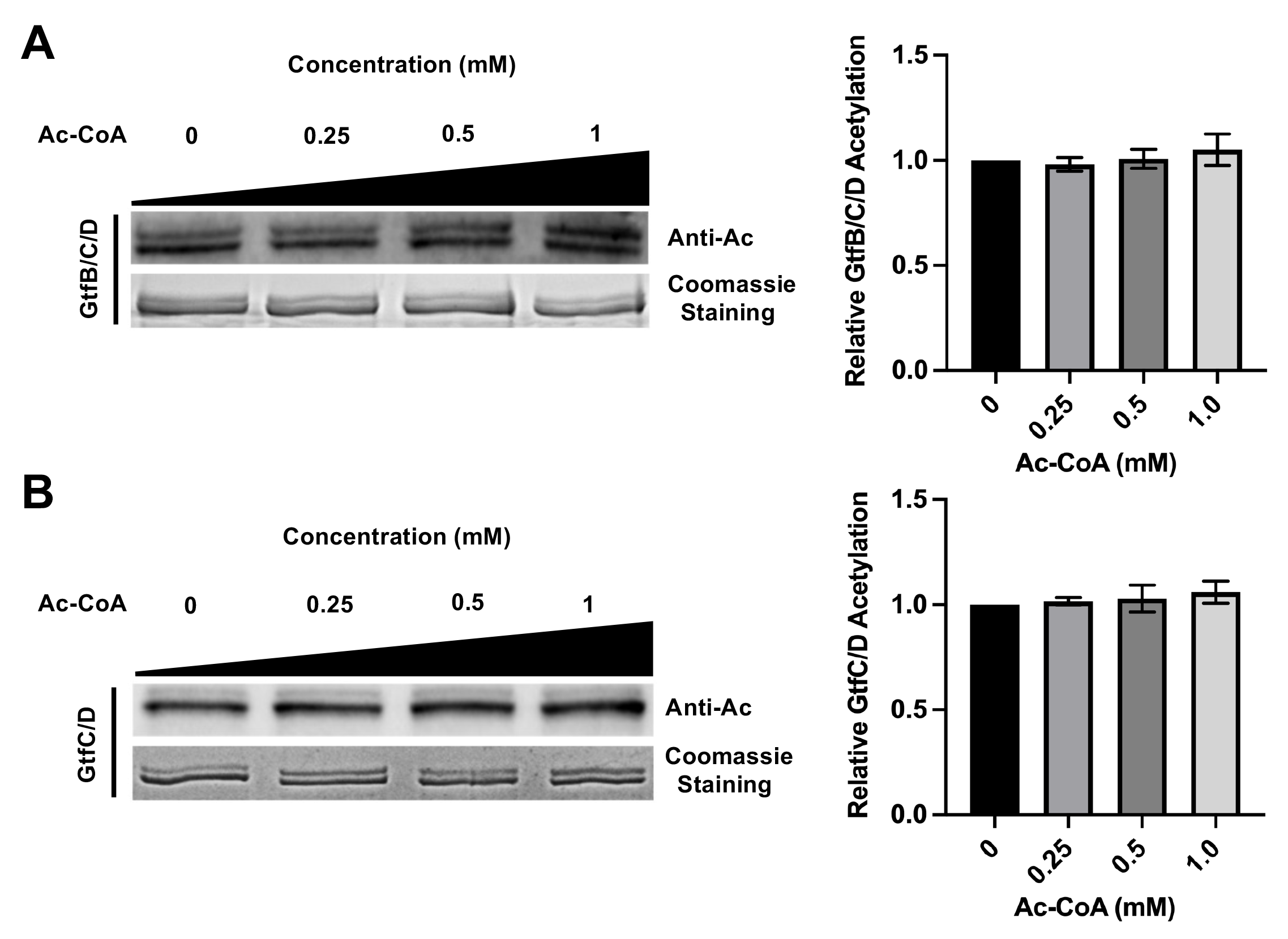

Supplement: S2 Fig — Coomassie staining and anti-acetyl lysine Western blotting analysis of GtfB/C/D (A) and GtfC/D (B) incubated with Ac-CoA as the acetyl donor at different concentrations for 3 h at 37°C. The band signals were quantified with Image J software and normalized to the control. Results are presented as mean ± SD (P > 0.05). (TIF) [file ppat.1010134.s002.tif]

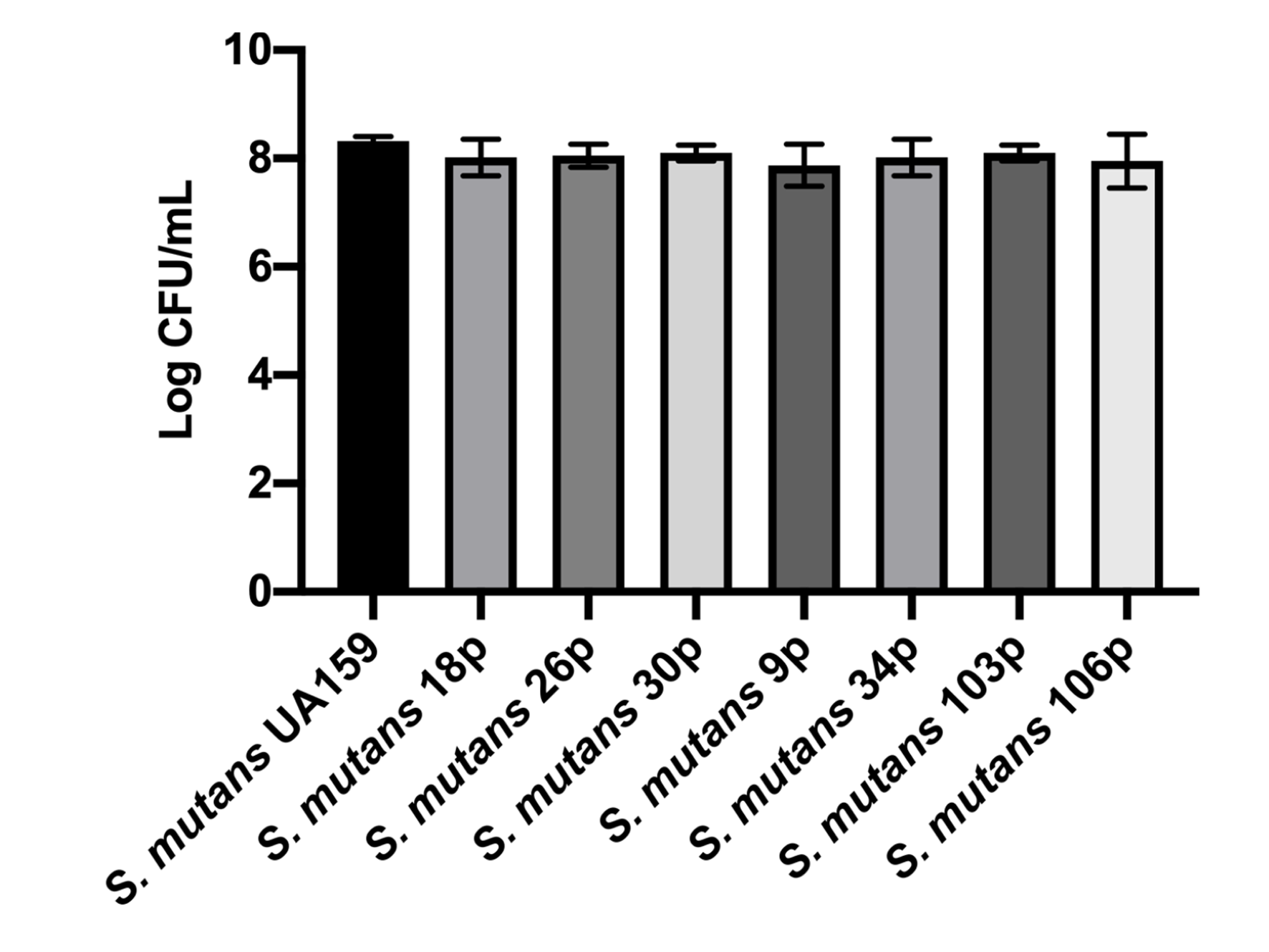

Supplement: S3 Fig — The cells from 24-hours biofilms were plated onto the BHI agar plate, incubated anaerobically for 48 h at 37°C, and the CFUs were counted. Results are presented as mean ± SD (P > 0.05). (TIF) [file ppat.1010134.s003.tif]
